# A rare case of tuberous sclerosis complex-associated renal cell carcinoma

**DOI:** 10.4102/sajr.v26i1.2406

**Published:** 2022-05-20

**Authors:** Humphrey Mapuranga, Bianca Douglas-Jones, Danelo du Plessis, Camilla E. le Roux, Christel du Buisson, Shahida Moosa

**Affiliations:** 1 Department of Radio-Diagnosis, Faculty of Medical Imaging and Clinical Oncology, Tygerberg Hospital, Stellenbosch University, Cape Town, South Africa; 2 Department of Medical Genetics, Division of Molecular Biology and Human Genetics, Tygerberg Hospital, Stellenbosch University, Cape Town, South Africa; 3 Department of Surgery, Division of Urology, Faculty of Medicine and Health Sciences, Tygerberg Hospital, Stellenbosch University, Cape Town, South Africa; 4 Department of Paediatrics and Child Health, Paediatric Nephrology, Faculty of Medicine and Health Sciences, Tygerberg Hospital, Stellenbosch University, Cape Town, South Africa

**Keywords:** tuberous sclerosis complex, renal cell carcinoma, paediatric, neuro-cutaneous, hamartomas

## Abstract

Renal cell carcinoma is rarely described in paediatric patients with tuberous sclerosis complex. This report describes a case of an 11-year-old male with tuberous sclerosis-associated renal cell carcinoma.

## Introduction

Tuberous sclerosis complex (TSC) is one of a large heterogeneous group of neurocutaneous syndromes with characteristic involvement of structures derived from embryologic neuro-ectoderm, and a prevalence of 1 in 6000–10 000 persons. The disease is characterised by slow-growing hamartomas with multisystem involvement being typical.

Tuberous sclerosis complex is an autosomal-dominant disorder, caused by heterozygous variants in one of the two genes: *TSC1*, which encodes for hamartin (located on chromosome 9q34), or *TSC2*, which encodes for tuberin (located on chromosome 16p13.3). Hamartin and tuberin act together as tumour suppressors and are components of the mammalian target of rapamycin (mTOR) signalling pathway.^1,2^

A clinical diagnosis of TSC is established if the individual has (1) two major clinical criteria, (2) one major and two or more minor criteria, or (3) the identification of a pathogenic variant in either *TSC1* or *TSC2* on genetic testing. Major criteria include multiple angiofibroma (≥ 3), cardiac rhabdomyoma, cortical dysplasias including tubers and white matter migration lines, hypomelanotic macules (≥ 3 mm of > 5 mm in diameter), lymphangioleiomyomatosis, retinal nodular hamartomas, shagreen patches, subependymal giant cell astrocytomas, subependymal nodules and ungual fibromas. Minor features include ‘confetti’ lesions on the skin, dental enamel pits, intraoral fibromas, multiple renal cysts, non-renal hamartomas and retinal achromic patches.

Angiomyolipomas (AMLs) are the most common renal lesions associated with TSC and are present in 75% – 80% of patients. Renal cell carcinomas (RCCs) are very rare in patients with TSC (1% – 4%) (2). Moreover, the average age of diagnosis of RCC in patients with TSC is 30 years, with the youngest TSC-associated RCC reported in a 6-month-old girl.^3^ The clear cell, papillary and chromophobe RCC subtypes have been described in association with TSC.^1,4^ Renal cysts and oncocytomas may also occur.^1,4^

This report describes a unique case of an 11-year-old boy with confirmed TSC-associated RCC and highlight his management.

## Patient presentation

An 11-year-old boy was referred to paediatric nephrology at Tygerberg Hospital with a clinical concern of hypertension following a seizure at his local hospital. He had a history of generalized tonic-clonic seizures from the age of three years. The seizure episodes were managed and controlled with sodium valproate. At presentation, he was fully awake and alert but persistently hypertensive with a blood pressure higher than the 95th percentile for his height, age and gender.

The patient was examined by a medical geneticist, and the clinical diagnosis of TSC was made, based on fulfillment of two major criteria: (1) > 3 hypomelanotic macules of > 5 mm in diameter, and (2) facial angiofibromas, in addition to further TSC-related features, like multiple ‘confetti’ skin lesions on his neck and chest and the clinical suspicion of multiple renal cysts. Based on the cystic kidneys, a contiguous gene deletion involving both *TSC2* and *PKD1* on chromosome 16p13.3 was considered as part of the differential molecular diagnosis, in addition to variants in *TSC1* or *TSC2*.

**FIGURE 1:**
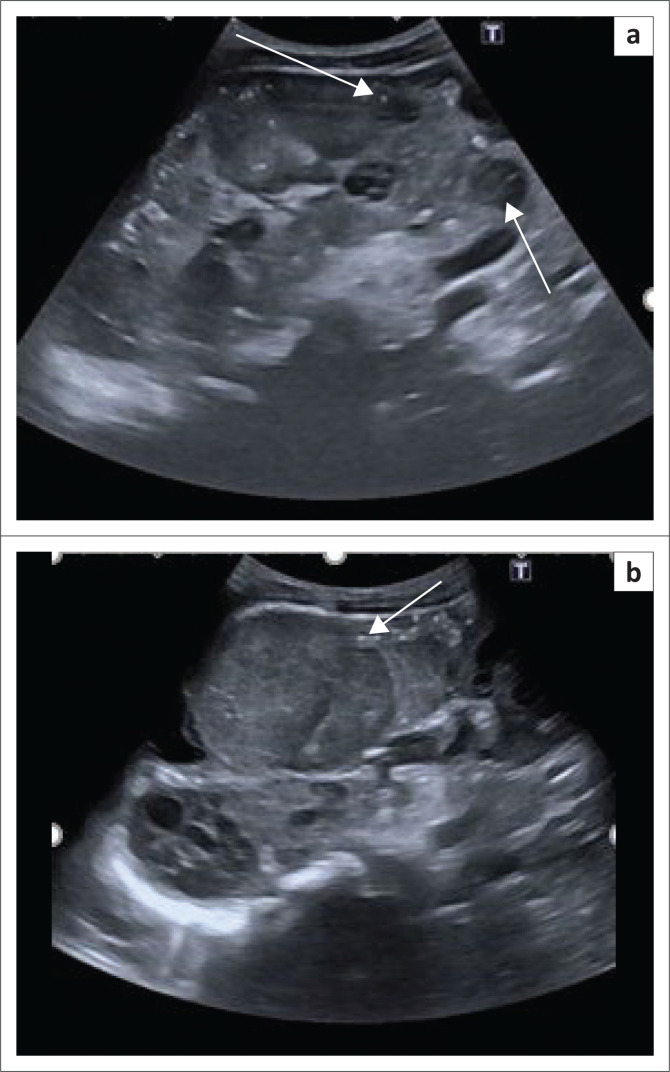
(a) Eleven-year-old male. Longitudinal greyscale ultrasound right kidney. Multiple markedly hypoechoic parenchymal masses were demonstrated (white arrows). (b) Longitudinal greyscale ultrasound left kidney. Large midpole mildly hypoechoic mass (white arrow).

Initial imaging evaluation at our institution included renal ultrasound, which demonstrated multiple bilateral mild to markedly hypoechoic cortical masses (Figure 1a and b). Magnetic resonance imaging (MRI) of the brain and abdomen was subsequently performed.

**FIGURE 2:**
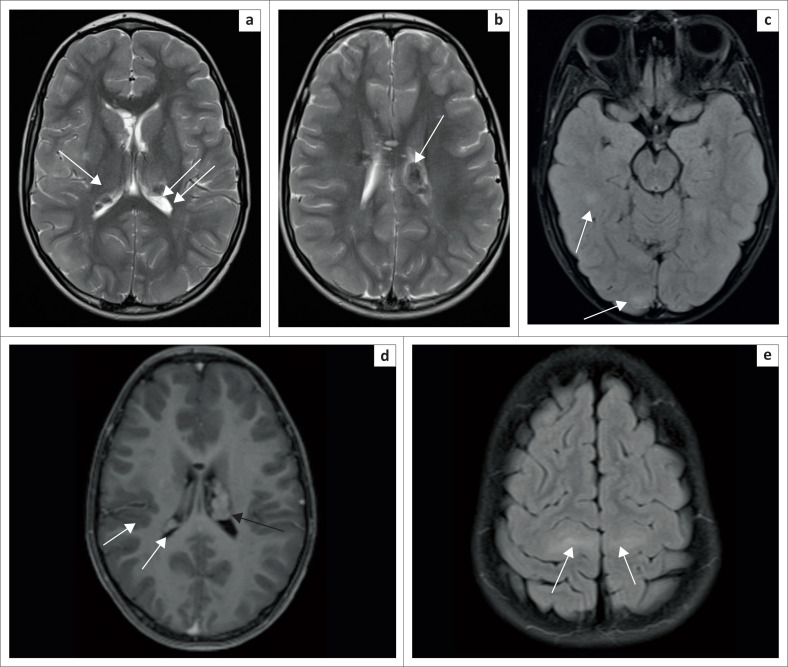
(a) Eleven-year-old male. T2W axial demonstrates numerous hypointense subependymal nodules (white arrows). (b) T2W axial demonstrated large subependymal giant cell astrocytoma (SEGA), with hypointense signal (white arrow). Additional subependymal nodule (black arrow). (c) Fluid inversion axial demonstrates hyperintense tubers (white arrows). (d) Axial T1-weighted precontrast image. Iso-intense subependymal nodule (white arrow). Subependymal giant cell astrocytoma (black arrow). (e) Axial fluid-attenuation inversion recovery (FLAIR) hyperintense white matter radial bands (white arrows).

Brain imaging demonstrated T2-weighted hypointense subependymal nodules (Figure 2a and b) with a subependymal giant cell astrocytoma (Figure 2a to d). Fluid-attenuated inversion recovery (FLAIR) demonstrated multiple hyperintense cortical tubers (Figure 2c) and hyperintense white matter radial bands (Figure 2e). No retinal hamartomas were identified.

**FIGURE 3:**
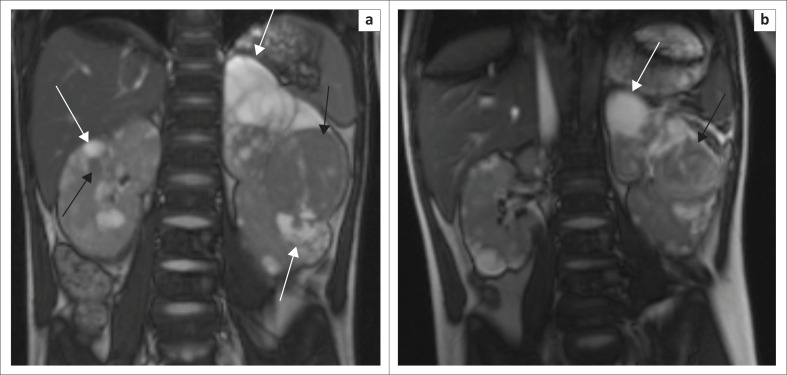
(a) Prior to treatment. T2-weighted coronal. Enlarged left kidney with multiple bilateral hyperintense (white arrows) and more hypointense masses (black arrows). (b) T2-weighted TRUFI coronal post-sirolimus and radiofrequency ablation of the left midpole renal cell carcinoma, with size reduction of left upper pole (white arrow) and midpole renal cell carcinoma ( black arrow).

**FIGURE 4:**
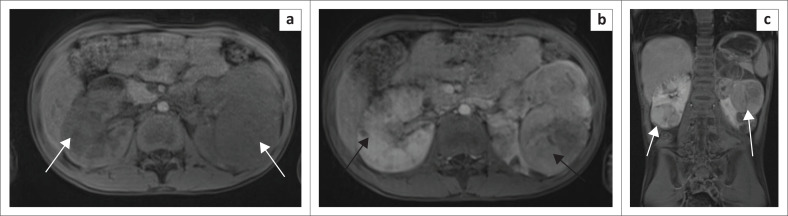
(a) T1-weighted axial abdomen demonstrates bilateral iso-intense renal mass lesions (white arrows). (b) T1-weighted axial abdomen post-contrast demonstrated enhancement of the bilateral renal mass lesions (black arrows). (c) T1-weighted coronal abdomen post-contrast demonstrates enhancement of bilateral renal mass lesions (white arrows).

Abdominal MRI demonstrated numerous bilateral renal cortical T2-weighted hypo to hyperintense (Figures 3a and b), T1-weighted hypointense mass lesions (Figure 4a). Fat suppression failed to demonstrate fat content (not shown). Post-contrast enhancement of bilateral renal mass lesions was seen. (Figure 4b and c). Time of flight MRI imaging of the abdominal aorta and renal arteries excluded renal artery stenosis (not shown). A radiological guided biopsy of the left midpole lesion was performed and confirmed tuberous sclerosis-associated RCC of the left kidney.

No concrete evidence of cardiac abnormalities, specifically rhabdomyomas, was found on echocardiogram.

## Testing, management and outcome

Genetic testing was performed in a stepwise manner. First, a chromosomal microarray was done and excluded the presence of a contiguous gene deletion on chromosome 16p13.3. The next step in testing was a TSC gene panel (Invitae, United States [US]), which included sequencing and deletion/duplication analysis of *TSC1* and *TSC2*. This showed a pathogenic variant in *TSC2* (c.5238_5255del; p.His1746_Arg1751del), which is a known pathogenic variant and definitively confirms the diagnosis.

The patient was started on sirolimus (a oral mTOR inhibitor) to treat his central nervous system (CNS) lesions. Due to the absence of demonstrable fat on MRI, a renal biopsy of the left midpole lesion was performed, which confirmed the diagnosis of tuberous sclerosis-associated RCC of the granular eosinophilic and macrocystic subtype.

The renal nephrometry score of the left midpole lesions was 11p, indicating a high complexity. Given the complexity and number of lesions, nephron sparing surgery was deemed too high a risk. We performed percutaneous ultrasound-guided microwave ablation (Emprint Ablation System, Medtronic) to treat the lesions in the left kidney, average wattage of 50 W and ablation time 6 min 30 s per lesion. Technical success was confirmed at post-ablation MRI (Figure 3b). Interval microwave ablation of the right renal lesions will be performed. The patient did not experience any post-ablation complications.

## Discussion

This patient had obvious features of TSC including, seizures due to underlying brain abnormalities, typical skin lesions, and hypertension as the presenting sign of underlying renal anomalies. With the identification of a pathogenic variant in *TSC2*, his condition was molecularly confirmed and enabled accurate genetic counselling. Up to two-third of patients with TSC have the condition due to a *de novo* (new) mutation. The risk to his offspring of inheriting the *TSC2* variant is 50%.

Renal lesions occur in 50% – 80% of patients with TSC, with AMLs being the most common (75% – 80%). Angiomyolipomas may be classified according to fat content into lipid rich, lipid poor and lipid absent.^5^ Renal cysts may also be identified in patients with TSC.^4^ While most RCCs occur sporadically, 3% – 5% are associated with hereditary syndromes, like Von-Hippel Lindau (VHL) disease, hereditary papillary RCC, and Birt–Hogg–Dubé (BHD) syndrome which is the most common. Renal cell carcinoma is very rare in TSC (< 3% of patients) and even rarer in the paediatric population (average age is 30 years).^4^

Treatment principles for these patients are cancer control, rather than cure, while preserving renal function. RCC develops metastatic potential as the size increases, and if individual tumour size can be kept below 3 cm – 7 cm in maximal diameter, the risk of metastases is estimated at less than 4%. Treatment should be nephron sparing, either surgical or using ablative therapy.^6,7^

Everolimus, an oral mTOR inhibitor, is effective in treating TSC-associated lesions, not only in the kidney, but also in the CNS; however, it is not recommended for the treatment of non-metastatic RCC.^8,9^

Renal cysts or polycystic kidney disease can develop in patients with TSC. As opposed to renal AMLs, renal cysts occur in younger children. Although renal cysts are generally asymptomatic, they can more frequently cause subsequent hypertension with rapid progression to end-stage renal failure.^10,12^ Thus, screening and treating hypertension and proteinuria, as well as preserving renal tissue, with the use of mTOR inhibitors, and sparing of renal tissue are advocated.^11,13^

## Conclusion

Microwave ablasion of selected cases of TSC-associated RCC, in conjunction with Everolimus, is a cornerstone of treatment for renal and central nervous system lesions in TSC.
